# Protein alignment based on higher order conditional random fields for template-based modeling

**DOI:** 10.1371/journal.pone.0197912

**Published:** 2018-06-01

**Authors:** Juan A. Morales-Cordovilla, Victoria Sanchez, Martin Ratajczak

**Affiliations:** 1 Dept. of Teoría de la Señal Telemática y Comunicaciones, Universidad de Granada, Granada, Spain; 2 Graz University of Technology, Signal Processing and Speech Communication Laboratory, Graz, Austria; Texas A&M University College Station, UNITED STATES

## Abstract

The query-template alignment of proteins is one of the most critical steps of template-based modeling methods used to predict the 3D structure of a query protein. This alignment can be interpreted as a temporal classification or structured prediction task and first order Conditional Random Fields have been proposed for protein alignment and proven to be rather successful. Some other popular structured prediction problems, such as speech or image classification, have gained from the use of higher order Conditional Random Fields due to the well known higher order correlations that exist between their labels and features. In this paper, we propose and describe the use of higher order Conditional Random Fields for query-template protein alignment. The experiments carried out on different public datasets validate our proposal, especially on distantly-related protein pairs which are the most difficult to align.

## 1 Introduction

Proteins carry out most of the work in living cells and their functions (structure, enzyme, messenger, …) are largely determined by their three dimensional (3D) structure which in turn is determined by the amino acid sequence [1]. Decoding the rules of these sequence–structure–function relationships is one of the most important open problems in science, not only in order to accelerate the understanding of the molecular functions in life, but also for protein-based biotechnologies and drug discovery [2]. Due to the high cost of the experimental methods (X-ray crystallography, nuclear magnetic resonance, …), the rate at which new protein sequences become available is much faster than the rate at which their structure and function are known [3]. Machine Learning techniques are bringing about new methods that fast and accurately predict the function [4, 5] and structure [6, 7] of proteins. In this paper we will focus on structure prediction methods which can be currently classified into one of the following two approaches [8]: 1) Free Modeling (FM) and 2) Template-Based Modeling (TBM).

Despite the great progress currently made on FM methods (mainly due to the incorporation of co-evolutionary information [2, 7, 9, 10]), FM remains computationally expensive (particularly for long-length proteins) and most of the servers for protein structure prediction (I-Tasser, Robetta, RaptorX, …) only use FM for those domains of the query protein that cannot be modeled by TBM. TBM methods basically consist of two steps [11]: 1) Find structurally related templates to the query protein by means of threading techniques (usually based on a ranking of the local alignment scores). 2) Build a 3D model of the query protein by means of a global alignment between the query and the selected template. This alignment is well known to perform poorly for distantly-related proteins (remote homologs) and in this paper we will focus on this problem.

Numerous methods have been proposed to solve the alignment problem. Just to name a few: the first proposed alignments based on substitution matrices [12, 13], PSI-Blast based on profile-sequence comparison [14], and HHAlign based on HMM sequence profiles [15]. However, most of these methods result in a poor performance when a query sequence is aligned to a distantly-related template. This is due to the fact that these methods heavily depend on the sequence profile [16] (they do not usually incorporate structural features such as secondary structure and solvent accessibility) and also because the scoring function (the measure of the alignment on a small region) is linear or has low expressive power (making it difficult to combine sequence and structural features). More information about the contribution to classification performance of the different types of features can be found in [15, 17, 18].

More recently, some of the most successful TBM methods have turned out to be those based on Conditional Random Fields (CRFs) (a discriminative graphical model used in Machine Learning [19]). To the best of our knowledge, CONTRAlign [20] was the first alignment method based on CRFs. It has local factors (scoring functions) based on linear combinations of simple features. Boosting-Threader [18] uses regression-tree factors to learn more complex relations between evolutionary and structural features and CNFPred [16] incorporates Neural Network factors and is embedded in the RaptorX server [21] (ranked among the top servers in the fully-automated blind test CAMEO [22]).

One of the main advantages of CRFs is that the scoring function not only represents the correlation among the features but also between the features and the labels at several positions. The scoring functions used so far in the above mentioned CRF-based alignment techniques are of order 1, i. e. they only represent the correlations between the current label (and features) and the previous one. However, the increase of computational power and the proposal of efficient inference algorithms [23–25] have recently allowed the use of Higher Order Conditional Random Fields (HO-CRFs) on different structured prediction tasks such as phoneme classification [26–28], and image recognition [29], obtaining a very good performance in such tasks due to the well known higher order correlations that exist in the speech (e. g. triphones) and image (e. g. superpixels) signals.

Our hypothesis is that the incorporation of a higher order scoring function will also be helpful for protein alignment since a larger region of the label-feature correlations could be learned. The main contribution of this paper is thus the development of a HO-CRF for protein alignment and the analysis of its performance on different datasets.

The rest of the paper is organized as follows. Sec. 2 is an overview of protein alignment with standard CRFs, Sec. 3 develops the extension of protein alignment with HO-CRFs, Sec. 4 describes the experimental setup including feature extraction and Sec. 5 analyzes the experimental results. We finish the paper with the most important conclusions and future work.

## 2 Alignment with standard CRF

**Fig 1:**
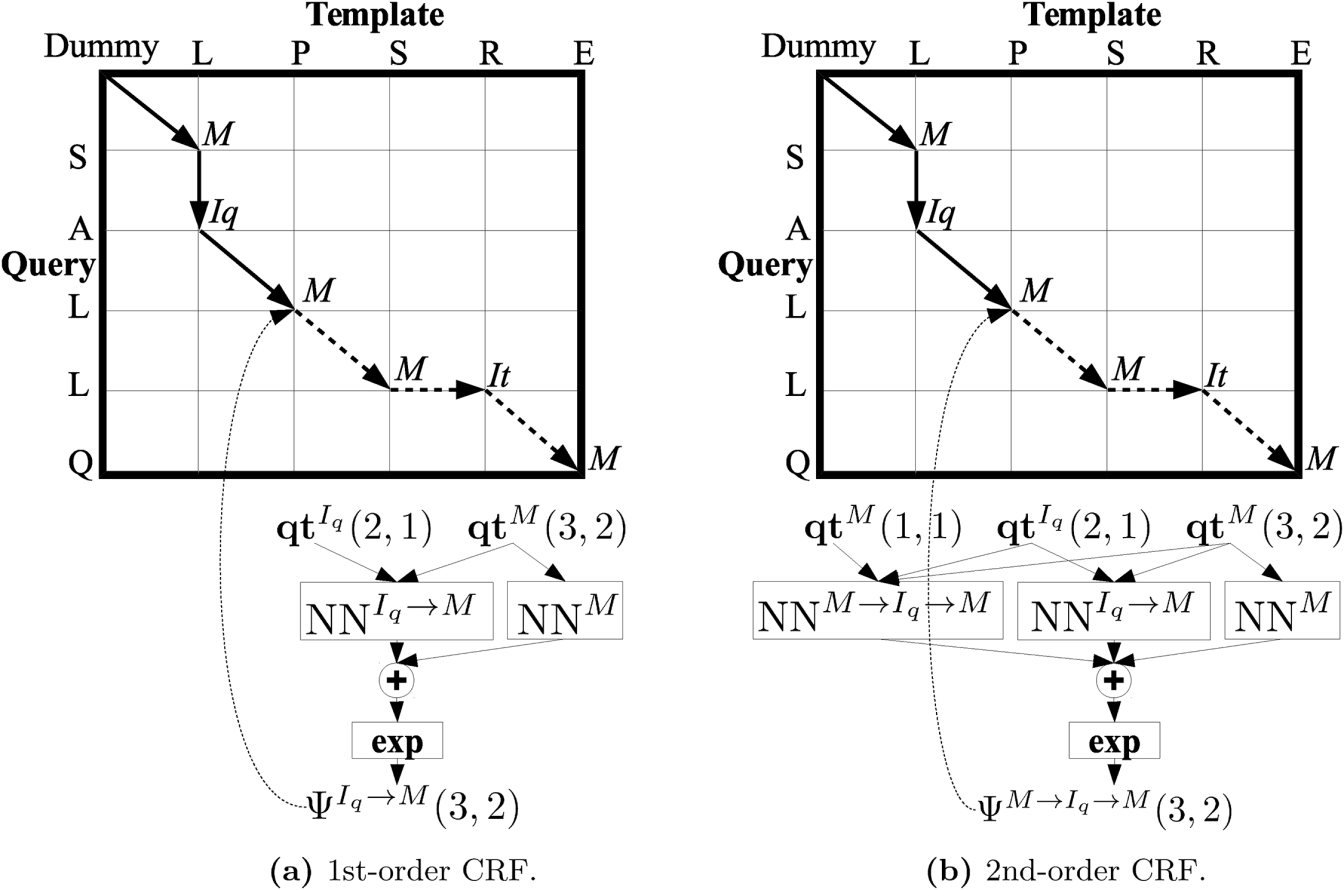
Alignment matrices and scoring functions Ψ of a 1st and 2nd-order CRF alignment for the label transitions *I*_*q*_ → *M* and *M* → *I*_*q*_ → *M*, respectively, at the alignment matrix position (3, 2). Ψ depends on log-factors outputs, Neural Networks (NNs) in our case, which in turn depend on the query and template feature vectors **qt**.

Before introducing the alignment method based on HO-CRF we will go over the standard alignment procedure based on 1st-order CRF presented at [8, 16, 18, 20]. As in [8], we will use the following notation. *L*_*q*_ and *L*_*t*_ are the lengths of the query and template proteins, respectively. *A* = *a*_1_ → *a*_2_… → *a*_*L*_*A*__ denotes an alignment of length *L*_*A*_ where each label or state *a*_*i*_ can be *M* (match), *I*_*q*_ (query insertion) or *I*_*t*_ (template insertion). One alignment can also be represented as a sequence of alignment positions (*x*_1_, *y*_1_) → (*x*_2_, *y*_2_)… → (*x*_*L*_*A*__, *y*_*L*_*A*__) where every position indicates the query and template residue indexes on the alignment matrix, respectively. As an example, Fig 1a shows an alignment which corresponds to the following label and position sequence: *A* = *M* → *I*_*q*_ → *M* → *M* → *I*_*t*_ → *M* = (1, 1) → (2, 1) → (3, 2) → (4, 3) → (4, 4) → (5, 5). The “Dummy” corner corresponds to the (0, 0) position.

### 2.1 Probability of one alignment

The order of a CRF is equal to the maximum number of previous labels considered. Taking into account a 1st-order CRF, let Ψ^*u* → *v*^(*x*_*i*_, *y*_*i*_) be the scoring function of a small alignment region with a label transition *u* → *v* at alignment position *i*. This function depends on the query-template feature vectors **qt** but for the sake of simplicity we omit them. The scoring function can be expressed as the exponential of the sum of a node log-factor *φ* and an edge log-factor *ϕ*:

$$\begin{array}{c} \Psi^{u\to v}\left(x_{i},y_{i}\right)=\exp\left(\varphi^{v}\left(x_{i},y_{i}\right)+\phi^{u\to v}\left(x_{i},y_{i}\right)\right) \end{array}$$ (1)

The log-factors relate different label transitions with the features and they can be any kind of functions. Here, they are Neural Networks as in [16] (for further details see Sec. 4.3). In total there are 12 (9 + 3) independent log-factors as we have 3 labels. Fig 1a depicts the log-factors (NNs) used to compute the scoring function of a label transition *I*_*q*_ → *M* at position (3, 2). We can see that the log-factor NN^*I*_*q*_ → *M*^ is fed by a final feature vector which is formed by the concatenation of 2 feature vectors: ${\text{qt}}^{I_{q}}\left(2,1\right)$ and **qt**^*M*^(3, 2) (see Sec. 4.1).

In a 1st-order CRF, the conditional probability of one alignment *A* given the query and template features (**qt**) and the parameter vector of all the log-factors (in our case NNs parameters ***θ***) can be expressed as follows:

$$\begin{array}{c} p\left(A|\text{qt},\theta \right)=p^{a_{1}\to a_{2}...\to a_{L_{A}}}=\frac{1}{Z}\prod_{i=2}^{L_{A}}\Psi^{a_{i-1}\to a_{i}}\left(x_{i},y_{i}\right) \end{array}$$ (2)

As can be seen, it is obtained as the product of the scoring functions normalized by a factor *Z* (known as partition function) that allows to properly compare different alignments. In the next section we will explain how to efficiently compute *Z*.

### 2.2 Forward-backward algorithm for Z function calculation

In this section we will show how to determine the partition function *Z* by means of the forward-backward algorithm [8]. Since we have 9 label patterns (*M* → *M*, *M* → *I*_*q*_, …, *I*_*t*_ → *I*_*t*_) we have to compute 3 forward and 3 backward terms (they correspond to the different suffixes and prefixes of these label patterns [24]) at every (*x*, *y*) position as follows:

$$\begin{array}{ccc} \alpha^{v}\left(x,y\right) & = & \sum_{u^{\prime }}\Psi^{u^{\prime }\to v}\left(x,y\right)\alpha^{u^{\prime }}\left(x_{p},y_{p}\right) \end{array}$$ (3)

$$\begin{array}{ccc} \beta^{u}\left(x,y\right) & = & \sum_{v^{\prime }}\Psi^{u\to v^{\prime }}\left(x_{n},y_{n}\right)\beta^{v^{\prime }}\left(x_{n},y_{n}\right) \end{array}$$ (4)

where *u*′ (or *v*′) of the summations goes over the 3 labels. The previous (*x*_*p*_, *y*_*p*_) (or the next (*x*_*n*_, *y*_*n*_)) position is determined by the current (*x*, *y*) position and the *v* (or *v*′) label of the scoring function Ψ as follows:

$$\begin{array}{c} \left(x_{p},y_{p}\right)=\left\{ \begin{array}{cc} \left(x-1,y-1\right) & \text{if}\;v=M\\ \left(x-1,y\right) & \text{if}\;v=I_{q}\\ \left(x,y-1\right) & \text{if}\;v=I_{t} \end{array}\right. \end{array}$$ (5)

For example, given (*x*, *y*) and $\Psi^{M\to I_{t}}$, then (*x*_*p*_, *y*_*p*_) = (*x*, *y* − 1) (and (*x*_*n*_, *y*_*n*_) = (*x*, *y* + 1)).

The partition function can then be computed as:

$$\begin{array}{c} Z=\sum_{v^{\prime }}\alpha^{v^{\prime }}\left(L_{q},L_{t}\right)=\sum_{u^{\prime }}\beta^{u^{\prime }}\left(0,0\right) \end{array}$$ (6)

where the initializations are: *α*^*v*^(0, 0) = 1 and *β*^*u*^(*L*_*q*_, *L*_*t*_) = 1. The corresponding derivative of these formulas with respect to each one of the parameters *θ* of the log-factors, i. e. $\frac{\partial \alpha^{v}\left(x,y\right)}{\partial \theta }$, $\frac{\partial \beta^{u}\left(x,y\right)}{\partial \theta }$ and $\frac{\partial Z}{\partial \theta }$, can be obtained as detailed in reference [8].

### 2.3 Training procedure

Given a set of *N* training alignments, the objective is to maximize the probability of observing them (maximum likelihood training [16]). In order to do so we are going to minimize the following loss function:

$$\begin{array}{c} l\left(\theta \right)=-\frac{1}{N}\sum_{n=1}^{N}\log p(A_{n}|{\text{qt}}_{n},\theta )+\frac{l_{2}}{L_{\theta }}\sum_{k=1}^{L_{\theta }}\theta_{k}^{2} \end{array}$$ (7)

where ***θ*** is the parameter vector of length *L*_***θ***_ (Sec. 2.1), log *p*(*A*_*n*_|**qt**_*n*_, ***θ***) is the log probability of one training alignment *A*_*n*_ (Eq 2) and *l*_2_ is a regularization term.

We use a stochastic gradient descent with learning rate *η* and momentum *γ* in order to minimize the loss function and to find the best parameter vector. We do not split the training set in batches, so we compute the average over the full training set of the loss function derivative *l*(***θ***) (Eq 7) with respect to every parameter in order to reestimate the new parameters at every training epoch. This derivative can be obtained by means of the derivative of the scoring function and of the partition function (see Sec. 2.2).

### 2.4 Test

In the test stage we implement the Viterbi algorithm, which consists of a maximization and a backtracking step, in order to find the optimal global alignment (*x*_1_, *y*_1_), (*x*_2_, *y*_2_), …(*x*_*L*_*A*__, *y*_*L*_*A*__) given the scoring functions Ψ^*u* → *v*^(*x*, *y*).

In the maximization step, we recursively compute 3 cumulative scores (*δ*^*v*^) and 3 optimal alignment subsequences of labels (Φ^*v*^) at each (*x*, *y*) position. We replace the sum operator of Eq (3) by the maximum operator as follows:

$$\begin{array}{c} \delta^{v}\left(x,y\right)=\max_{u^{\prime }\to v}\Psi^{u^{\prime }\to v}\left(x,y\right)\delta^{u^{\prime }}\left(x_{p},y_{p}\right) \end{array}$$ (8)

$$\begin{array}{c} \Phi^{v}\left(x,y\right)={\text{argmax}}_{u^{\prime }\to v}\Psi^{u^{\prime }\to v}\left(x,y\right)\delta^{u^{\prime }}\left(x_{p},y_{p}\right) \end{array}$$ (9)

Every Φ^*v*^(*x*, *y*) records an optimal alignment subsequence of the type u* → *v*.

In the backtracking step, the alignment starts at the corner position (*x*_*L*_*A*__, *y*_*L*_*A*__) = (*L*_*q*_, *L*_*t*_) and at the “move” *a*_*L*_*A*__ with the highest cumulative score at that position: *a*_*L*_*A*__ = argmax_*v*′_ *δ*^*v*′^(*x*_*L*_*A*__, *y*_*L*_*A*__). Then we apply the following recursion: given a current position (*x*_*i*_, *y*_*i*_) and a current move *a*_*i*_, the previous position (*x*_*i*−1_, *y*_*i*−1_) can be determined by *a*_*i*_ using Eq (5). The previous move *a*_*i*−1_ is equal to u* where $u^{*}\to a_{i}=\Phi^{a_{i}}\left(x_{i},y_{i}\right)$. The recursion finishes when (*x*_*i*_, *y*_*i*_) is out of the alignment matrix.

## 3 Alignment with a higher order CRF

We will show now how to extend the 1st-order CRF alignment, described in Sec. 2, to a 2nd-order CRF alignment building on the HO-CRF formulation presented at [24, 30]. The extension to higher orders or even to a sparse CRF alignment could be derived in a similar way.

### 3.1 Probability of one alignment

The scoring function of a 2nd-order CRF alignment can be expressed as:

$$\begin{array}{c} \Psi^{t\to u\to v}\left(x_{i},y_{i}\right)=\exp\left(\varphi^{v}\left(x_{i},y_{i}\right)+\phi^{u\to v}\left(x_{i},y_{i}\right)+\chi^{t\to u\to v}\left(x_{i},y_{i}\right)\right) \end{array}$$ (10)

If we compare it with the 1st-order CRF scoring function (Sec. 2.1) we can now observe a new 2nd-order log-factor (*χ*) that models a longer alignment subsequence (see Fig 1a and 1b where the alignment subsequences of Ψ are indicated by continuous lines). This increases the expressive power of our CRF at the expense of an increase in the number of parameters. Compared to the 1st-order CRF, not only the number of log-factors (39 = 27 + 9 + 3) has increased but also the new 2nd-order log-factors are more complex as they are fed by larger size feature vectors (see Sec. 4.1). Nonetheless, we will see in Sec. 5.1 that this increase in the number of parameters does not produce overfitting.

Then, in a 2nd-order CRF, the probability of one alignment can be now expressed as follows:

$$\begin{array}{c} p\left(A|\text{qt},\theta \right)=p^{a_{1}\to a_{2}...\to a_{L_{A}}}=\frac{1}{Z}\prod_{i=3}^{L_{A}}\Psi^{a_{i-2}\to a_{i-1}\to a_{i}}\left(x_{i},y_{i}\right) \end{array}$$ (11)

### 3.2 Forward-backward algorithm

Since we now have 27 label patterns (*M* → *M* → *M*, *M* → *M* → *I*_*q*_, …, *I*_*t*_ → *I*_*t*_ → *I*_*t*_) we have to compute 9 forward and 9 backward terms (corresponding to the different suffixes and prefixes of these label patterns [24]) at every (*x*, *y*) position as follows:

$$\begin{array}{c} \alpha^{u\to v}\left(x,y\right)=\sum_{t^{\prime }}\Psi^{t^{\prime }\to u\to v}\left(x,y\right)\alpha^{t^{\prime }\to u}\left(x_{p},y_{p}\right) \end{array}$$ (12)

$$\begin{array}{c} \beta^{t\to u}\left(x,y\right)=\sum_{v^{\prime }}\Psi^{t\to u\to v^{\prime }}\left(x_{n},y_{n}\right)\beta^{u\to v^{\prime }}\left(x_{n},y_{n}\right) \end{array}$$ (13)

The previous (*x*_*p*_, *y*_*p*_) (or the next (*x*_*n*_, *y*_*n*_)) position is determined by the current (*x*, *y*) position and the *v* (or *v*′) label of the scoring function as in Sec. 2.2. The partition function can now be computed as:

$$\begin{array}{c} Z=\sum_{u^{\prime },v^{\prime }}\alpha^{u^{\prime }\to v^{\prime }}\left(L_{q},L_{t}\right)=\sum_{t^{\prime },u^{\prime }}\beta^{t^{\prime }\to u^{\prime }}\left(0,0\right) \end{array}$$ (14)

where the initializations are: *α*^*u* → *v*^(0, 0) = 1 and *β*^*t* → *u*^(*L*_*q*_, *L*_*t*_) = 1. The corresponding derivative of these formulas with respect to each parameter *θ* of the log-factors can be obtained in a similar way as in reference [8].

### 3.3 Training

The loss function used for the 2nd-order CRF is the same one as that employed in the 1st-order CRF, see Eq (7), but now using the 2nd-order CRF probability (Eq 11) instead. The same stochastic gradient descent method is also used to find the minimum.

The derivative with respect to every parameter of the loss function requires the derivatives of the partition function and of the forward terms at the corner position (see Sec. 3.2). In order to do so, we need to compute the forward and the scoring function derivatives at all positions which is quite memory demanding and time consuming due to the high number of parameters and positions (specially in the 2nd-order CRF). In order to reduce computational resources, it has been implemented in a diagonal recursive way, i. e. we compute all the derivatives of one alignment matrix diagonal at every iteration. The memory is reduced by maintaining only the previous diagonals required to compute the current diagonal. The computational time is reduced because by vectorization we parallelize the computations of diagonal elements which are independent of each other.

### 3.4 Test

A similar Viterbi algorithm to that described in Sec. 2.4 has been implemented for the HO-CRF but now in the maximization step, we find the 9 cumulative scores (*δ*^*u* → *v*^) and 9 optimal alignment subsequences Φ^*u* → *v*^(*x*, *y*) at each (*x*, *y*) position. We then replace the sum operator of Eq (12) by the maximum operator as follows:

$$\begin{array}{c} \delta^{u\to v}\left(x,y\right)=\max_{t^{\prime }\to u\to v}\Psi^{t^{\prime }\to u\to v}\left(x,y\right)\delta^{t^{\prime }\to u}\left(x_{p},y_{p}\right) \end{array}$$ (15)

$$\begin{array}{c} \Phi^{u\to v}\left(x,y\right)=\underset{t^{\prime }\to u\to v}{\text{argmax}}\Psi^{t^{\prime }\to u\to v}\left(x,y\right)\delta^{t^{\prime }\to u}\left(x_{p},y_{p}\right) \end{array}$$ (16)

Every Φ^*u*→*v*^(*x*, *y*) now records an optimal alignment subsequence of the type t* → *u* → *v*.

In the backtracking step, the alignment starts at the corner position (*x*_*L*_*A*__, *y*_*L*_*A*__) = (*L*_*q*_, *L*_*t*_) and at the “move” with the highest cumulative score at that position: *a*_*L*_*A*_−1_ → *a*_*L*_*A*__ = argmax_*u*′→*v*′_ *δ*^*u*′→*v*′^(*x*_*L*_*A*__, *y*_*L*_*A*__). Then we apply the following recursion: given a current position (*x*_*i*_, *y*_*i*_) and a current move *a*_*i*−1_ → *a*_*i*_ the previous position (*x*_*i*−1_, *y*_*i*−1_) can be determined by *a*_*i*_ using Eq (5). The previous move *a*_*i*−2_ → *a*_*i*−1_ is equal to t* → *a*_*i*−1_ where $t^{*}\to a_{i-1}\to a_{i}=\Phi^{a_{i-1}\to a_{i}}\left(x_{i},y_{i}\right)$. The recursion finishes when (*x*_*i*_, *y*_*i*_) is out of the alignment matrix.

## 4 Experimental setup

### 4.1 Features

As in [16, 18], we extract evolutionary and structural features using publicly available software. In particular, we use the program *buildFeature* of CNFPred to compile all the evolutionary and structural features in one file (.tgt and .tpl for the query and template proteins, respectively). In turn, *buildFeature* calls several external programs that are described next. PSI-BLAST [14] on the Non Redundant databases NR70 and NR90 to generate the position specific scoring matrix *PSSM* for the template and the position specific frequency matrix *PSFM* for the query. HHPred together with HHMake also on these databases to obtain the HMM query and template profiles. PDB-Tool [31] for the 3-class Secondary Structure (SS3) and Solvent Accessibility (SA) values of the template and PSIPRED [32] together with RaptorX-ACC [33] for SS3 and SA state probabilities estimation of the query. Finally, DISOPRED [34] is used to set to 0 all structural features when disordered regions are detected.

The features used for each CRF label are then the following ones:

- Features for a match label (*M*): sequence profile similarity (*PSSM*-*PSFM*) [18], sequence similarity using BLOSUM50 [13] and SS3 and SA match score [18] of the query-template residues at the current position.
- Features for a query-insertion label (*I*_*q*_): context hydropathy count [20], Relative Solvent Accessibility (RSA) and SS3 values of the template residue at the current position.
- Features for a template-insertion label (*I*_*t*_): context hydropathy count, RSA estimation and SS3 estimation of the query residue at the current position.

The RSA estimation of a query residue at position *x* is computed based on the probabilities of being at a buried (B), medium (M) or exposed (E) state at that position as follows:

$$\begin{array}{c} \text{RSA}(x)=10p(B(x))+42p(M(x))+100p(E(x)) \end{array}$$ (17)

where the cutoff values 10 and 42 are chosen as in RaptorX. Compared to [16, 18], we are using a smaller number of features and all of them are mean and variance normalized in order to accelerate the training convergence. The features corresponding to positions out of the alignment matrix are set to zero.

We have just described the composition of the feature vector associated to each type of CRF label. Then the final log-factor feature vector has to be obtained. As an example, let us consider the log-factor NN^*M*→*I*_*q*_→*M*^ of Fig 1b. It is fed by the feature vectors **qt**^*M*^(1, 1), ${\text{qt}}^{I_{q}}\left(2,1\right)$ and **qt**^*M*^(3, 2) that are concatenated obtaining a final log-factor feature vector of size 11 = 4 + 3 + 4.

### 4.2 Datasets

We build our dataset from the LINDAHL benchmark (see [35] for description and availability on the Web). LINDAHL contains 7438 positive pairs, i. e. protein pairs which share the same fold (structurally-related proteins). From these pairs, we randomly select 50 (12, 7, 31) for training, 50 (12, 2, 36) for validation and the rest 7338 (1622, 2121, 3595) for our in-house test set called POSLINDAHL. In parenthesis we indicate the number of pairs resulting at the Family, Superfamily and Fold levels. The pairs selected for the training and validation sets are limited to pairs in which query and template sequence lengths are smaller than 100 amino acids. We establish this limitation due to the training complexity of the 2nd-Order CRF alignment (Sec. 3.3 and 4.3). Each protein pair of the POSLINDAHL set shares around 16 (22, 15, 13)% of sequence identity (SeqID).

In addition to the POSLINDAHL test set, we will evaluate our algorithms with two more public datasets: PROSUP ([36] for description and availability) which consists of 127 pairs (16% of SeqID) and SALIGN (test-set) ([37] for description and availability) which consists of 200 pairs (20% of SeqID). In Sec. 5.2 we will give more details about the difficulty of these datasets by means of the TM-Score.

In all cases, the structure alignment tool DeepAlign [38] provides us the reference alignment. As we are interested in global alignment, only the region between the first and the last aligned residues provided by this tool are employed in our experiments. For the test sets, the [minimum, average, maximum] of the sequence lengths and the length difference of the pairs (|*L*_*q*_ − *L*_*t*_|/*max*(*L*_*q*_, *L*_*t*_)%) are [4, 137, 527] and 15% for POSLINDAHL, [46, 181, 491] and 11% for PROSUP, and [117, 278, 741] 10% for SALIGN.

### 4.3 CRF parameters

We describe next how the values of the 1st and 2nd-order CRF parameters are optimized by means of our training and validation sets. 21 factors (out of the 39 log-factors) are a Neural Network with one hidden layer of 12 neurons without bias as in [16], the remainder are just constant values which do not depend on the features. These constant log-factors correspond to the label transitions that appear in the training set with a probability smaller than 0.002 and for which there is not enough data to train them properly. In this way we reduce the number of parameters to be trained which are now *L*_***θ***_ = 901 and 2941 for the 1st and 2nd-order CRFs, respectively. For the sake of simplicity we use the same values for the stochastic gradient descent parameters in the 1st and 2nd-order CRFs. They are: *l*_2_ = 0.001, *η* = 0.01 and *γ* = 0.5.

**Fig 2:**
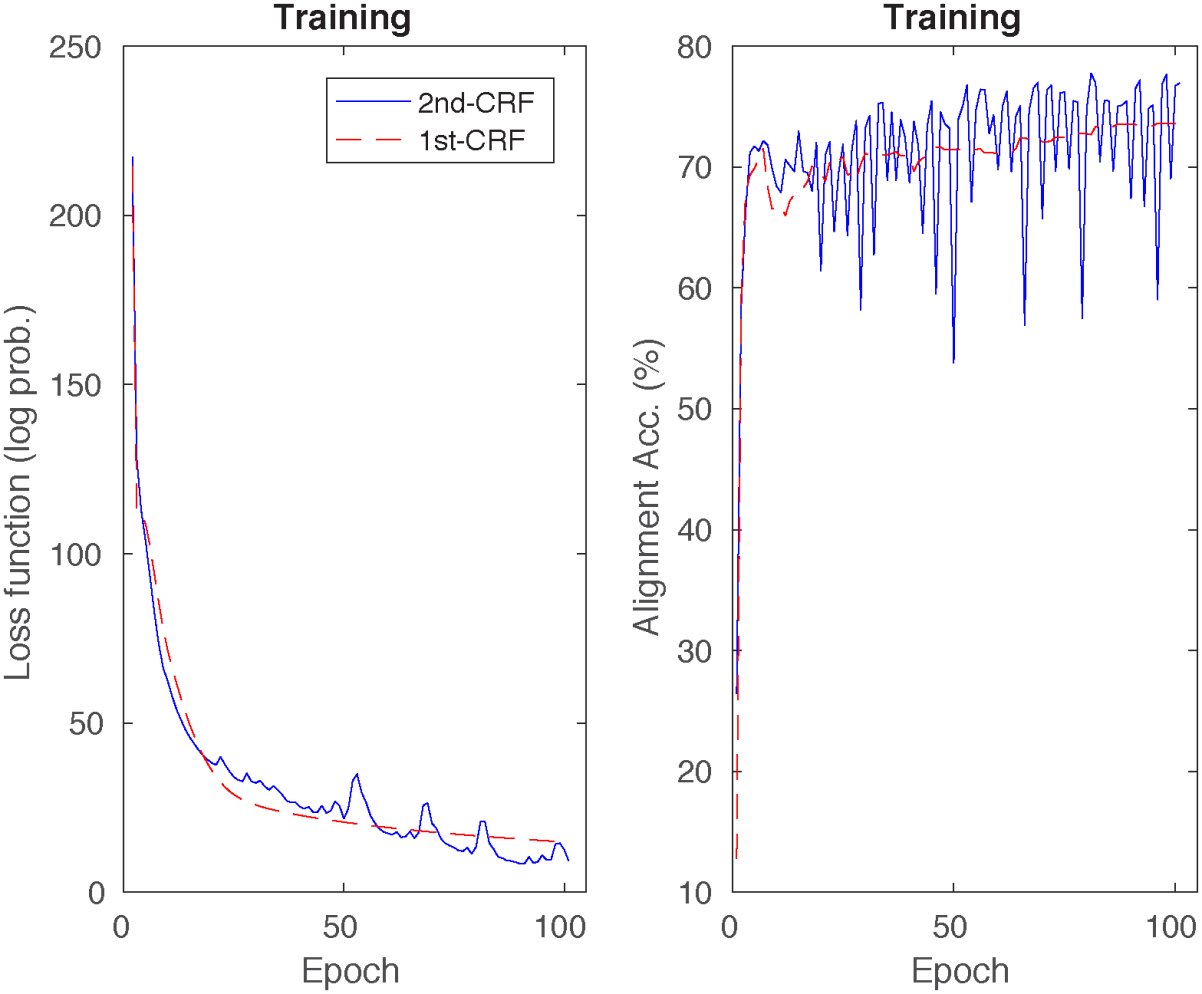
Loss function and alignment accuracies of the 1st and 2nd-order CRFs on the training set at every epoch.

Fig 2 shows the loss function, and the alignment accuracy of the 1st and 2nd-order CRFs at every epoch of the training set. Each epoch of the 1st and 2nd-order CRFs takes in our computer (Intel(R) Core(TM) i7-4790 CPU 3.60GHz) around 1 and 8 minutes, respectively. The 1st and 2nd-order CRF curves cannot be directly compared as they can converge at different speed, however we can see that, in general, the 2nd-CRF performs better than the 1st-CRF. By means of the validation sets we selected our best 1st and 2nd-order CRFs models from epochs 93 and 99, respectively.

### 4.4 Baseline software tools

We compare our HO-CRF based alignment with the following alignment methods:

- The state of the art CNFPred [16] based on Conditional Neural Fields of order 1. Compared to our 1st-order CRF, CNFPred employs a wider variety of features such as Secondary Structure with 8 states (SS8), a position specific gap probability, context-specific potentials (of size 11) and other strategies such as geometrical constraints in the Viterbi search [39]. Moreover, it has been trained on a larger dataset of 1010 protein pairs and with a more effective loss function that directly maximizes the TM-Score.
- The state of the art alignment method HHAlign [40] based on HMM profiles alignment [15]. We obtained the HMM profiles with the program HHPred of *buildFeature* (see Sec. 4.1) and then we ran HHAlign with the options ‘-loc -mact 0.1’ (denoted as loc in future comparisons) and ‘-glob’ (denoted as glob). These two options provided the best results among other tried.
- CONTRAlign [20], the first CRF alignment method proposed and based on linear factors, with its default hydropathy options. Compared to our 1st-order CRF, both use the same global Viterbi algorithm (Sec. 2.4). The only difference lies in the scoring functions (Eq 1) and, consequently, in the log-factors which are more complex in 1st-order CRF. For example, the edge log-factor *ϕ*^*M*→*M*^(*x*, *y*) of 1st-order CRF is an NN that depends on the current (x, y) structural and evolutionary features while in CONTRAlign it is a constant value which does not depend on the current features.
- The global alignment NWAlign of MATLAB (with its default parameters) based on the Needleman-Wunsch [12] algorithm and the BLOSUM50 matrix [13].

## 5 Experimental results and discussion

### 5.1 Results on alignment

Table 1 shows the reference dependent accuracies (%) [16, 18], defined as the percentage of correctly aligned query amino acids judged by the reference alignments, for several alignment methods on different test sets. A query amino acid is correctly aligned when the template amino acid associated by the method is the same as the template amino acid associated by DeepAlign. For the POSLINDAHL set we show the average and the Family, Superfamily and Fold (Fa., Su., Fo) level accuracies. 1st and 2nd CRF-Align, HHAlign (loc) and (glob), and CNFPred derive their features from the same.tgt and .tpl files mentioned in Sec. 4.1 in order to have a fairer comparison.

**Table 1:** Reference dependent alignment accuracy (%) for different methods on the test sets.

Method	POSLINDAHL (Fa., Su., Fo.)	SALIGN	PROSUP
2nd-CRF-Align	**51.92** (71, **48**, **37**)	67.60	64.68
CNFPred	50.36 (**73**, **48**, 30)	**73.26**	**68.04**
1st-CRF-Align	47.11 (68, 43, 31)	63.42	59.35
HHAlign (loc)	44.70 (72, 46, 17)	68.58	64.26
HHAlign (glob)	38.26 (70, 37, 7)	67.15	61.19
CONTRAlign	42.54 (64, 38, 26)	56.37	54.37
NWAlign	37.34 (56, 32, 24)	46.48	45.96

We can observe that the two best techniques are 2nd-CRF-Align and CNFPred. On one hand, 2nd-CRF-Align performs best on POSLINDAHL (51.92%), particularly at Fold level (37%) which is the most difficult set (composed of distantly-related proteins). On the other hand, CNFPred obtains the best results on SALIGN and PROSUP. If we now take all the 7665 tested proteins (POSLINDAHL + SALING + PROSUP), we find that the percentage of cases in which 2nd-CRF-Align outperforms CNFPred is 51.96% (where 219 equal results are discarded). In order to test the significance of this result we have applied a Wilcoxon signed-rank test at the 0.05 significance level. The result of the test allows us to reject the null hypothesis (i. e., that the difference between 2nd-CRF-Align and CNFPred results is zero median) with a p-value = 4.9 × 10^−12^ < 0.05, and to infer that the already mentioned percentage, in which 2nd-CRF-Align outperforms CNFPred, is statistically significant.

If we carry out a similar comparison with HHAlign (loc) we obtain that the percentage of cases in which 2nd-CRF-Align outperforms HHAlign (loc) is 67.25% (p-value = 0). The poor performance of HHAlign (loc) at Fold level (17%) can be explained due to the fact that, as we mentioned in the Introduction, HHAlign heavily depends on the sequence profile which becomes sparse and not well estimated at this level. In addition, HHAlign (loc), compared to CNFPred and 2nd-CRF-Align, does not incorporate structural features which are very useful at Fold level.

The comparison between 1st-CRF-Align and 2nd-CRF-Align confirms our initial hypothesis that the incorporation of a higher order scoring function can help in the alignment. Although 1st-CRF-Align shares the same CRFs architecture as CNFPred, the additional features and strategies introduced by CNFPred (and mentioned in Sec. 4.4) can explain why 1st-CRF-Align performs worse in general. In fact, if we increase our training set to 100 protein pairs and we consider a context of size 7 for the match label features, the results obtained by 1st-CRF-Align are now 49.70 (69, 45, 35) for POSLINDAHL, 65.50 for SALIGN and 64.26 for PROSUP, i. e. an absolute improvement of almost 3% on average. All of this suggests that an increase in feature and training sizes would improve the results of 1st-CRF-Align and, consequently, of 2nd-CRF-Align as well. However, we have avoided this increase on the 2nd-CRF-Align technique for two reasons. The first one is that the computational cost of the training stage would be very high (see Sec. 4.3) and the second reason is that, despite the small training set (50 proteins), our 2nd-CRF-Align technique is not overfitted to the training set (see Sec. 3.1) and obtains competitive results compared to the other state of the art methods.

### 5.2 Results on structure prediction

The results of the CRF based techniques of Table 1 could be biased towards the reference alignment (DeepAlign) as the other methods are not trained on it. In order to avoid this possible advantage, as also done in [16], we will as well evaluate the 3D-model of the query protein predicted from the query-template alignment. We will use the software MODELLER [41] which takes both the alignment and the template PDB file and estimates the query PDB file. The quality of this estimation is measured by the TM-Score [42]. This score compares the estimated with the true query PDB file and it has a value ranging from 0 to 1, indicating the worst and best model quality, respectively. The true query PDB file employed in the comparison is the one comprised between the first and the last aligned residues of the reference alignment provided by PDB-Tool [31]. Table 2 shows the cumulative TM-Scores (or reference independent alignment accuracies) for different methods and datasets.

**Table 2:** Reference independent alignment accuracy for different methods on the test sets (measured by cumulative TM-Score of the query 3D model).

Method	POSLINDAHL (Fa., Su., Fo.)	SALIGN	PROSUP
2nd-CRF-Align	**1041** (**965**, 912, **1245**)	129	72.2
CNFPred	1012 (964, **913**, 1159)	**137**	**72.3**
1st-CRF-Align	962 (933, 841, 1113)	123	66.3
HHAlign (loc)	885 (948, 865, 841)	129	69.2
HHAlign (glob)	780 (930, 775, 634)	124	65.3
CONTRAlign	894 (901, 772, 1009)	111	62.5
NWAlign	875 (857, 741, 1027)	103	59.2
DeepAlign (oracle)	1052 (1078, 1216, 1774)	153	86.8

Analyzing Table 2 we can reach the same conclusions as those derived from Table 1. It is interesting to point out that now the 2nd-CRF-Align technique, apart from obtaining outstanding results at Fold level, it performs as CNFPred on the POSLINDAHL (at both Family and Superfamily levels) and PROSUP datasets. From a similar Wilcoxon test we can infer that 2nd-CRF-Align outperforms CNFPred and HHAlign (loc) in 57.63% (p-value = 8.7 × 10^−107^) and 69.38% (p-value = 0) of the cases, respectively.

The performance comparison between the 1st and 2nd-CRF-Align methods finally corroborate the benefits of using a HO-CRF for the alignment. The last row of the table (DeepAlign (oracle)) shows the results that we would obtain if the reference structure alignment was used and it gives an idea of the maximum performance that we could achieve in 3D structure prediction by alignment methods. If instead of obtaining the cumulative TM-Score of DeepAlign (oracle), we compute the average per protein TM-Score, we obtain 0.57 (0.66, 0.57, 0.49) for POSLINDAHL, 0.76 for SALIGN and 0.68 for PROSUP. These numbers, together with the sequence identity percentage indicated in Sec. 4.2, characterize the difficulty of the different sets.

These results allow us to reach the following conclusion: when the protein pairs are closely related, as on SALING (TM-Score = 0.76), 2nd-CRF-Align gives competitive results but CNFPred is better. However for distantly-related pairs, as at the Fold level in POSLINDAHL (TM-Score = 0.49), the best predictor is 2nd-CRF-Align.

## 6 Conclusions

In this work, we have described how to carry out query-template alignment of proteins using a Higher Order Conditional Random Field (HO-CRF). We have based our proposal on previous developments regarding the use of first order CRFs for protein alignment [16, 18, 20] and the formulation of HO-CRFs [23, 24, 30]. The experimental results on different public datasets of structurally-related proteins have shown that there exist higher order correlations between the features and the labels of an alignment and that the use of HO-CRF makes sense, especially when the proteins of the pair are more distantly related (i. e. the most difficult tasks). Although this paper has focused on 2nd-order CRF alignment, the extension to higher order alignments would be direct. However, this extension would require an increase in the training size to avoid overfitting and, consequently, a GPU implementation in order to accelerate HO-CRFs training. In addition, the exploitation of sparsity aspects and the incorporation of co-evolution information (as in MRFAlign [10]) are suggested as promising research directions for future work.

## References

[1] Anfinsen CB. Principles that govern the folding of protein chains. Science. 1973;181(4096):223–230. doi: 10.1126/science.181.4096.223 4124164 10.1126/science.181.4096.223

[2] Service RF. This protein designer aims to revolutionize medicines and materials. Science. 2016;.

[3] Jo T, Hou J, Eickholt J, Cheng J. Improving Protein Fold Recognition by Deep Learning Networks. Scientific Reports. 2015;5 doi: 10.1038/srep17573 10.1038/srep17573 PMC4669437 26634993

[4] Bernardes JS, Pedreira CE. A review of protein function prediction under machine learning perspective. Recent Patents on Biotechnology. 2013;7(2):122–141. doi: 10.2174/18722083113079990006 23848274 10.2174/18722083113079990006

[5] Clares JD, Sánchez V, Peinado AM, Morales-Cordovilla JA, Iribar C, Peinado JM. Improved Image Based Protein Representations with Application to Membrane Protein Type Prediction. In: IEEE International Conference on Telecommunications and Signal Processing; 2017.

[6] Cheng J, Tegge AN, Baldi P. Machine Learning Methods for Protein Structure Prediction. IEEE Reviews in Biomedical Engineering. 2008;1:41–49. doi: 10.1109/RBME.2008.2008239 22274898 10.1109/RBME.2008.2008239

[7] Wang S, Sun S, Li Z, Zhang R, Xu J. Accurate De Novo Prediction of Protein Contact Map by Ultra-Deep Learning Model. PLoS Computational Biology. 2017;13(1):e1005324 doi: 10.1371/journal.pcbi.1005324 28056090 10.1371/journal.pcbi.1005324 PMC5249242

[8] Ma J. Protein Structure Prediction by Protein Alignments. Toyota Technological Institute at Chicago; 2015.

[9] Marks DS, Colwell LJ, Sheridan R, Hopf TA, Pagnani A, Zecchina R, et al Protein 3D Structure Computed from Evolutionary Sequence Variation. PLoS ONE. 2011;6(12):e28766 doi: 10.1371/journal.pone.0028766 22163331 10.1371/journal.pone.0028766 PMC3233603

[10] Ma J, Wang S, Wang Z, Xu J. MRFalign: Protein Homology Detection through Alignment of Markov Random Fields. PLoS Computational Biology. 2014;10(3):e1003500 doi: 10.1371/journal.pcbi.1003500 24675572 10.1371/journal.pcbi.1003500 PMC3967925

[11] Wang C, Zhang H, Zheng WM, Xu D, Zhu J, Wang B, et al FALCON@home: a high-throughput protein structure prediction server based on remote homologue recognition. Bioinformatics. 2016;32(3):462–464. doi: 10.1093/bioinformatics/btv581 26454278 10.1093/bioinformatics/btv581 PMC4804767

[12] Needleman SB, Wunsch CD. A general method applicable to the search for similarities in the amino acid sequence of two proteins. Journal of Molecular Biology. 1970;48(3):443–453. doi: 10.1016/0022-2836(70)90057-4 5420325 10.1016/0022-2836(70)90057-4

[13] Henikoff S, Henikoff JG. Amino acid substitution matrices from protein blocks. Proc Natl Acad Sci USA. 1992;89(22):10915–10919. doi: 10.1073/pnas.89.22.10915 1438297 10.1073/pnas.89.22.10915 PMC50453

[14] Altschul SF, Madden TL, Schaeffer AA, Zhang J, Zhang Z, Miller W, et al Gapped BLAST and PSI-BLAST: a new generation of protein database search programs. Nucleic Acids Research. 1997;25(17):3389–3402. doi: 10.1093/nar/25.17.3389 9254694 10.1093/nar/25.17.3389 PMC146917

[15] Söding J. Protein homology detection by HMM-HMM comparison. Bioinformatics. 2005;21(7):951–960. doi: 10.1093/bioinformatics/bti125 15531603 10.1093/bioinformatics/bti125

[16] Ma J, Peng J, Wang S, Xu J. A conditional neural fields model for protein threading. Bioinformatics. 2012;28(12):59–66. doi: 10.1093/bioinformatics/bts213 10.1093/bioinformatics/bts213 PMC3371845 22689779

[17] Cheng J, Baldi P. A machine learning information retrieval approach to protein fold recognition. Bioinformatics. 2006;22(12):1456–1463. doi: 10.1093/bioinformatics/btl102 16547073 10.1093/bioinformatics/btl102

[18] Peng J, Xu J. Boosting Protein Threading Accuracy. In: International Conference on Research in Computational Molecular Biology (RECOMB); 2009. p. 31–45. 10.1007/978-3-642-02008-7_3 PMC3325114 22506254

[19] Lafferty J, McCallum A, Pereira FCN. Conditional Random Fields: Probabilistic Models for Segmenting and Labeling Sequence Data. In: International Conference on Machine Learning (ICML); 2001. p. 282–289.

[20] Do CB, Gross SS, Batzoglou S. CONTRAlign: discriminative training for protein sequence alignment. International Conference on Computational Molecular Biology (RECOMB) Lecture Notes in Computer Science Springer. 2006;3909.

[21] Källberg M, Wang H, Wang S, Peng J, Wang Z, Lu H, et al Template-based protein structure modeling using the RaptorX web server. Nature Protocols. 2012;7:1511–1522. doi: 10.1038/nprot.2012.085 22814390 10.1038/nprot.2012.085 PMC4730388

[22] Haas J, Roth S, Arnold K, Kiefer F, Schmidt T, Bordoli L, et al The Protein Model Portal-a comprehensive resource for protein structure and model information. Database. 2013;bat031 doi: 10.1093/database/bat031 23624946 10.1093/database/bat031 PMC3889916

[23] Qian X, Jiang X, Zhang Q, Huang X, Wu L. Sparse Higher Order Conditional Random Fields for improved sequence labeling. In: Neural Information Processing Systems (NIPS); 2009. p. 849–856.

[24] Ye N, Lee WS, Chieu HL, Wu D. Conditional random fields with high-order features for sequence labeling. In: Neural Information Processing Systems (NIPS); 2009. p. 2196–2204.

[25] Ye N. Probabilistic learning: Sparsity and non-decomposable losses. Department of Computer Science. National University of Singapore; 2013.

[26] Ratajczak M, Tschiatschek S, Pernkopf F. Neural Higher-Order Factors in Conditional Random Fields for Phoneme Classification. In: Interspeech; 2015. p. 2137–2141.

[27] Ratajczak M, Tschiatschek S, Pernkopf F. Virtual Adversarial Training Applied to Neural Higher-Order Factors for Phone Classification. In: Interspeech; 2016. p. 2756–2760.

[28] Ratajczak M, Tschiatschek S, Pernkopf F. Frame and Segment Level Recurrent Neural Networks for Phone Classification. In: Interspeech; 2017.

[29] Arnab A, Jayasumana S, Zheng S, Torr P. Higher Order Conditional Random Fields in Deep Neural Networks. In: European Conference on Computer Vision; 2016. p. 524–540.

[30] Ratajczak M, Tschiatschek S, Pernkopf F. Structured Regularizer for Neural Higher-Order Sequence Models. In: European Conference on Machine Learning (ECML); 2015. p. 168–183.

[31] Wang S, Zheng WM. CLePAPS: fast pair alignment of protein structures based on conformational letters. J Bioinform Comput Biol. 2008;6(2):347–66. doi: 10.1142/S0219720008003461 18464327 10.1142/s0219720008003461

[32] Jones DT. Protein Secondary Structure Prediction Based on Position-specific Scoring Matrices. Journal of Molecular Biology Elsevier. 1999;292:195–202. doi: 10.1006/jmbi.1999.3091 10.1006/jmbi.1999.3091 10493868

[33] Wang Z, Zhao F, Peng J, Xu J. Protein 8-class secondary structure prediction using conditional neural fields. Proteomics. 2011;11(19):3786–3792. doi: 10.1002/pmic.201100196 21805636 10.1002/pmic.201100196 PMC3341732

[34] Ward JJ, McGuffin LJ, Bryson K, Buxton BF, Jones DT. The DISOPRED server for the prediction of protein disorder. Bioinformatics. 2004;20(13):2138–2139. doi: 10.1093/bioinformatics/bth195 15044227 10.1093/bioinformatics/bth195

[35] Lindahl E, Elofsson A. Identification of related proteins on family, superfamily and fold level. Journal of Molecular Biology. 2000;295(3):613–625. Dataset available from: http://iris.rnet.missouri.edu/dnfold/data/lindahl_data doi: 10.1006/jmbi.1999.3377 10623551 10.1006/jmbi.1999.3377

[36] Lackner P, Koppensteiner WA, Sippl MJ, Domingues FS. ProSup: a refined tool for protein structure alignment. Protein Engneering. 2000;13(11):745–752. Dataset available from: https://www.came.sbg.ac.at/archive_came_services/Benchmark/HellM.pairs doi: 10.1093/protein/13.11.745 10.1093/protein/13.11.745 11161105

[37] Marti-Renom MA, Madhusudhan MS, Sali A. Alignment of protein sequences by their profiles. Protein Science. 2004;13(4):1071–1087. Dataset available from: https://salilab.org/suppmat/mamr03 doi: 10.1110/ps.03379804 15044736 10.1110/ps.03379804 PMC2280052

[38] Wang S, Ma J, Peng J, Xu J. Protein structure alignment beyond spatial proximity. Scientific Reports. 2013;3:1448 doi: 10.1038/srep01448 23486213 10.1038/srep01448 PMC3596798

[39] Peng J, Xu J. Low-homology protein threading. Bioinformatics. 2010;26(12):294–300. doi: 10.1093/bioinformatics/btq192 10.1093/bioinformatics/btq192 PMC2881377 20529920

[40] Alva V, Nam SZ, Söding J, Lupas AN. The MPI bioinformatics Toolkit as an integrative platform for advanced protein sequence and structure analysis. Nucleic Acids Research. 2016;44:410–415. doi: 10.1093/nar/gkw348 10.1093/nar/gkw348 PMC4987908 27131380

[41] Webb B, Sali A. Comparative Protein Structure Modeling Using MODELLER. Current Protocols in Bioinformatics John Wiley & Sons, Inc. 2016;54:5.6.1–5.6.37. doi: 10.1002/cpbi.3 10.1002/cpbi.3 PMC5031415 27322406

[42] Zhang Y, Skolnick J. Scoring function for automated assessment of protein structure template quality. Proteins. 2004;57(4):702–710. doi: 10.1002/prot.20264 15476259 10.1002/prot.20264

